# Altered Basal Autophagy Affects Extracellular Vesicle Release in Cells of Lagotto Romagnolo Dogs With a Variant *ATG4D*

**DOI:** 10.1177/0300985820959243

**Published:** 2020-10-05

**Authors:** Pernilla Syrjä, Mari Palviainen, Tarja Jokinen, Kaisa Kyöstilä, Hannes Lohi, Petra Roosje, Linda Anderegg, Tosso Leeb, Antti Sukura, Eeva-Liisa Eskelinen

**Affiliations:** 1 3835 University of Helsinki, Helsinki, Finland; 2 Folkhälsan Research Center, Helsinki, Finland; 3 27210 University of Bern, Bern, Switzerland; 4 8058 University of Turku, Turku, Finland

**Keywords:** basal autophagy, extracellular vesicles, canine, ATG4D, immunoelectron microscopy, NTA, mass spectrometry, disease model

## Abstract

Lagotto Romagnolo breed dogs develop a progressive neurological disease with intracellular vacuolar storage when homozygous for a variant in the autophagy-related gene 4D (*ATG4D*). A lysosomal enzyme deficiency has not been proven in this disease, despite its overlapping morphology with lysosomal storage diseases. Instead, basal autophagy was altered in fibroblasts from affected dogs. The aim of this study was to clarify the origin of the limiting membrane of the accumulating vacuoles and determine whether altered basal autophagy affects the extracellular release of vesicles in cells from diseased dogs. When assessed by immunoelectron microscopy, the membrane of the cytoplasmic vacuoles in affected tissues contained ATG4D, markers for autolysosomes (microtubule-associated protein 1A/B light chain 3 and lysosome-associated membrane protein 2) and for recycling endosomes (transferrin receptor 2), indicating that the vacuoles are hybrid organelles between endocytic and autophagic pathways. Ultracentrifugation, nanoparticle tracking analysis, and mass spectrometry were used to analyze the vesicles released from cultured fibroblasts of affected and control dogs. The amount of extracellular vesicles (EVs) released from affected fibroblasts was significantly increased during basal conditions in comparison to controls. This difference disappeared during starvation. The basal EV proteome of affected cells was enriched with cytosolic, endoplasmic reticulum, and mitochondrial proteins. Heat shock proteins and chaperones, some of which are known substrates of basal autophagy, were identified among the proteins unique to EVs of affected cells. An increased release of extracellular vesicles may serve as a compensatory mechanism in disposal of intracellular proteins during dysfunctional basal autophagy in this spontaneous disease.

Extracellular vesicles (EVs) are a diverse group of membrane-bound particles released by several cell types into the surrounding extracellular space. These vesicles have various intracellular origins and are produced via several different mechanisms.^12,28^ The function and content of EVs has been a field of intense research since the discovery that EVs can transfer RNA between cells^29^ and that spread of toxic and/or infectious proteins can be propagated through the release and uptake of EVs.^3,16,27^ EVs also function in intercellular signaling and may contain disease-specific content and potential biomarkers, as increased EV release is one compensatory disposal mechanism for misfolded or aggregated protein.^8,16,27^ The classification of EVs based on size and release mechanism is not strict, but continuously evolving. The term EVs comprises both exosomes, which are small, <100-nm intraluminal vesicles (ILVs) released from the endosomal compartment through fusion of multivesicular bodies with the plasma membrane, and larger >100-nm microvesicles directly budding from the plasma membrane.^12,28^ Larger vesicles, which expose phosphatidylserine and form through apoptosis, are also shed as EVs.^12,28^ In addition, it was recently shown that the autophagic pathway affects EV biogenesis, as EVs may display both autophagosomal and exosomal proteins.^12,16^ Amphisomes, hybrid organelles between the endosomal and the autophagosomal compartments, also release several nonvesicular nanoparticles co-isolating with EVs, such as DNA and histones.^12^

Within the cell, macroautophagy encloses aggregated protein and damaged organelles from the cytoplasm into a double-membrane-bound vesicle, the autophagosome, and shuttles the content to the lysosome for degradation.^17,18^ A transient organelle, the autolysosome, is formed by fusion between the autophagosome and the lysosome. The autolysosomal content is thereafter released into the cytoplasm as nutrients or as material for biosynthetic reactions, or it is exocytosed through fusion of the autolysosome with the plasma membrane.^17,18^ When cells enclose extracellular material into a cytoplasmic membrane-bound vesicle through endocytosis, the content enters the endolysosomal pathway, with alternating endpoints similar to autophagy. The endosomal content can be delivered back to the plasma membrane and released extracellularly through recycling endosomes or through multivesicular bodies. It can also be digested after fusion with the lysosome. The autophagic and the endolysosomal pathways intersect at the level of multivesicular bodies, as autophagosomes may fuse with multivesicular bodies to form amphisomes.^10,14^ The mechanisms determining and regulating whether the vesicular content is released extracellularly, or degraded intracellularly, are largely unknown and may differ depending on the nutrient conditions of the cell.^19,32^

Lagotto Romagnolo dogs homozygous for the mutant allele at a missense variant of the autophagy-related gene *ATG4D* (c1288G>A; p. Ala430Thr) develop progressive neurological decline and a histopathological phenotype characterized by intracellular accumulation of membrane-bound vacuoles in various cells.^13,26^ Cells of affected dogs show altered basal autophagy, but functional starvation-induced autophagy. Accumulation of single membrane-bound vacuoles in the cell has long been considered the morphological hallmark of lysosomal storage diseases, a group of degenerative progressive diseases caused by defective production, function, or targeting of lysosomal degradative enzymes or lysosomal proteins. Several lysosomal storage diseases affecting dogs have been used in disease modeling as they share the genetic background and morphologic phenotype with similar human diseases. However, it has also been shown that disruption in the endolysosomal flux^19^ or in the release of autophagic vacuolar compartments^4^ may lead to accumulation of intracellular membrane-bound vacuoles, despite normal lysosomal function. So far, the origin of the vacuolar limiting membrane and the fate of the accumulating vacuoles in affected Lagotto Romagnolo dogs are still unknown, as neither a lysosomal defect nor accumulation of autophagosomes has been linked to the disease. The aim of this study was to elucidate the origin of the limiting membrane of the accumulating vacuoles and to test the hypothesis that the extracellular release of vesicles is altered during basal autophagy in the affected cells.

## Materials and Methods

### Ethics Statement

The affected dogs were privately owned pets, undergoing euthanasia and autopsy at request of the owner, due to progressive neurological signs. The control dogs underwent surgery for various medical reasons (Table 1) with the owners’ consent. Sampling was performed according to clinical standards approved by the Animal Ethics Committee at the State Provincial Office of Southern Finland (Permit: ESAVI/6054/04.10.03/2012).

**Table 1.** Demographic Data Regarding the Lagotto Romagnolo Dogs From Which Dermal Fibroblasts Were Obtained for the Study.

Case	Group	Sex	Age	Main clinical finding
1	Affected	Female	5 years	Hemorrhagic gastroenteritis, neurological decline^a^, ataxia
2	Affected	Male	5 years	Neurological decline^a^, ataxia
3	Affected	Female	7 years	Neurological decline^a^, ataxia
4	Control	Female	4 years	Ovariohysterectomy
5	Control	Female	5 years	Mammary tumor
6	Control	Male	8 years	Skin tumor

^a^ Neurological decline included episodic nystagmus and behavioral changes such as restlessness, depression, and aggression.

### Material

Fibroblasts from skin biopsies of 3 clinically affected Lagotto Romagnolo dogs (cases 1–3), genetically confirmed to be homozygous for the *ATG4D^mut/mut^* genotype (c.1288G>A; p.Ala430Thr), were included in the study. One dog underwent postmortem examination at the University of Bern, Switzerland (case 3), and 2 dogs at the University of Helsinki, Finland (cases 1 and 2). Fibroblasts obtained from the ventral skin incision during routine surgery of 3 clinically healthy Lagotto Romagnolo dogs (cases 4–6) were used as control material. Two control dogs were heterozygous (cases 4 and 6) and 1 dog was wild type (case 5) for the *ATG4D* variant. Demographic data of the affected and control dogs included in the study are presented in Table 1.

### Immunoelectron Microscopy

Tissue samples of the dorsal root ganglion (DRG) and pancreas of one affected dog (case 1) were obtained within 1 hour after death. Tissues were fixed for 2 hours in 4% paraformaldehyde, then transferred to 2% paraformaldehyde, until embedded into Lowicryl K4M for immunoelectron microscopy (IEM). Briefly, grids with ultrathin tissue sections were blocked with 5% bovine serum albumin (BSA; Sigma-Aldrich), 0.5% fish skin gelatin (G7765, Sigma-Aldrich CAS 9000-70-8), and 1% fetal bovine serum (FBS; 10500056 Gibco) in NaPO_4_ buffer, pH 7.4.

The grids were then incubated with primary antibodies against microtubule-associated protein 1A/B light chain 3 (LC3; Abcam ab48394 rabbit polyclonal) as a marker for autophagosomal membranes, transferrin-receptor 2 (TFR2; ThermoFischer PA5-42732 rabbit polyclonal) as a marker for endosomal membranes, lysosomal-associated membrane protein-2 (LAMP2; LSBio 3144 mouse monoclonal) as a marker for lysosomal membranes, and ATG4D (SAB1301447; Sigma Aldrich rabbit polyclonal) for 2 hours at room temperature. For determining nonspecific staining density, control sections without primary antibody (for LAMP2) or incubated with a rabbit-isotype control antibody (Rabbit IgG polyclonal isotype control Abcam ab37415, for ATG4D, LC3, and TFR) were included. After washing, a 20-minute incubation with protein-A-conjugated 10-nm gold or gold-conjugated goat-anti mouse secondary antibody was performed. Sections were washed, post-fixed for 5 minutes with 5% glutaraldehyde, and post-stained with uranyl acetate and lead citrate.

Samples were viewed with a Jeol Jem-1400 (Jeol Ltd) electron microscope equipped with a Gatan Orius SC1000B bottom mounted CCD camera (Gatan Inc) at 80 kV. The raw gold labelling density of membranous and nonmembranous areas in the cells was quantified using ImageJ software (Fiji, by Wayne Rasband, National Institute of Mental Health, Bethesda, MD). A minimum of 50 gold particles, or all particles in 10 fields of view at 10 000× magnification, were recorded as either membranous or nonmembranous. Gold particles within 15 nm of a membrane were considered membranous. The total membranous versus nonmembranous area in the pictures were measured. The specificity of the label was calculated for each antibody, according to guidelines for quantitative assessment of specificity in IEM.^15^ Briefly, the specific labelling density was counted as the total membranous gold density (dots/μm^2^) minus the nonspecific membranous labelling density noted in sections incubated without a primary antibody or with the isotype control antibody (ie, D(s) = D (+) − D (−)). For antibodies detecting both a membranous and a cytoplasmic protein (LC3, ATG4D), the specificity was also assessed using the χ^2^ test comparing membranous versus nonmembranous gold label distribution with that of the isotype control.

### Analysis of Extracellular Vesicles

Fibroblasts from 3 affected and 3 control Lagotto Romagnolo dogs were seeded at equal cell density, during second or third passage, into two 175 cm^2^ flasks each and cultured for 48 hours in high-glucose Dulbecco’s modified Eagle medium (DMEM; 41965-039 Gibco) supplemented with 10% fetal bovine serum (FBS; 10500056 Gibco) and penicillin-streptomycin (PenStrep; 15140122 Gibco). The cells were then washed 3 times with phosphate-buffered saline (PBS) and the medium changed into 25 ml DMEM supplemented with 10% EV-free fetal bovine serum (EV core facility, University of Helsinki; FBS centrifuged at 110 000*g* for 18 hours) and penicillin-streptomycin per flask. Cells were grown into semiconfluency for 72 hours, and after visual control of cellular integrity, the medium was harvested from affected and control cells by decanting into sterile Falcon tubes.

The cells were washed thrice with PBS and subjected to a 2-hour starvation in Earl’s Balanced Salt Solution (EBSS; 24010043 Gibco). The conditioned EBSS was harvested after the starvation. The total harvested medium (50 ml) from each culture was immediately cooled, and centrifuged at 2500*g* and +4 °C for 30 minutes. The supernatant with EVs was frozen at −80 °C. EVs were enriched through ultracentrifugation at 110 000*g* for 2 hours. The pellet was resuspended in 1 ml of Dulbecco’s phosphate-buffered saline (DPBS) buffer and further ultracentrifuged at 110 000*g* for 90 minutes. The final EV pellet was resuspended in 100 µl of (DPBS). For quality control, one sample from each dog was viewed using negative staining by transmission electron microscopy. EVs were loaded on 200 mesh pioloform- and carbon-coated glow-discharged copper grids. Samples were fixed with 2% PFA (Electron Microscopy Sciences) in 0.1 M NaPO_4_ buffer (pH 7.0), stained with 2% neutral uranyl acetate, embedded in methyl cellulose uranyl acetate mixture (1.8/0.4%), and viewed with transmission electron microscopy using Tecnai 12 (FEI Company, Eindhoven, The Netherlands) operating at 80 kV. Images were taken with Gatan Orius SC 1000B CCD-camera (Gatan Inc).

The EV concentration and size distribution was determined by nanoparticle tracking analysis (NTA) with Nanosight model LM14 (Malvern Panalytical) equipped with blue (404 nm, 70 mW) laser and SCMOS camera. The samples were diluted in DPBS and five 30-second videos were recorded using camera level 14. The data were analyzed using NTA software 3.0 with the detection threshold 1 and screen gain 5. Comparisons between the concentration of EVs in media from 3 affected and 3 control fibroblasts in one experiment under basal conditions (FM = full media) and starvation (ST = starvation) were performed. The mean EV concentration in each sample was determined as an average of 5 measurements using NTA. The size distribution of EVs in affected and control samples was compared using mean percentiles for cutoffs at 100 nm, 150 nm, and 250 nm in diameter. The differences in mean EV concentration and in mean % of EVs smaller than 100 nm, 150 nm, and 250 nm between affected and control were tested for statistical significance using the linear mixed model method for dependent samples. Differences with *P* < .05 were considered statistically significant.

### Extracellular Vesicle Proteomics

**Figure 1.**
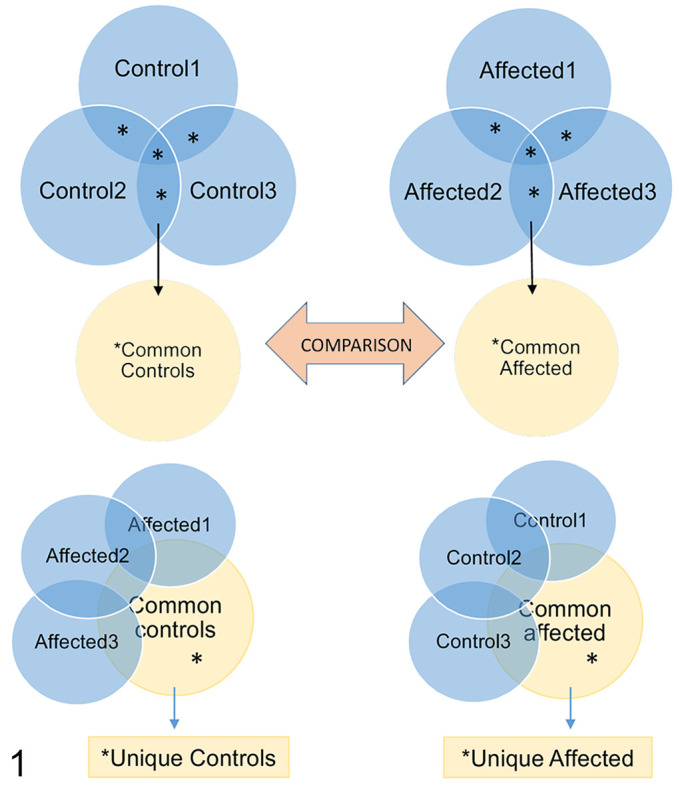
Overview of the grouping and workflow regarding the analysis of the extracellular vesicle (EV) proteomes. The common EV proteome of controls (Common controls; asterisks in top left panel) was defined as proteins identified in EVs of 2 or more control dogs. The common EV proteome of affected cells (Common affected; asterisks in top right panel) was defined as proteins identified in 2 or more affected dogs. The Common affected and Common control proteomes were compared. Proteins unique to control EVs (asterisks in lower left panel) and unique to affected EVs (asterisks in lower right panel) were defined as proteins present in the common proteome of the group but absent in the comparison group.

The proteomes of EVs harvested from 50 ml media of affected and control fibroblasts during basal conditions and after 2-hour starvation were analyzed by mass spectrometry. Briefly, the whole proteome of EVs in 50 ml medium of equally seeded, semiconfluent fibroblasts from affected and control Lagotto Romagnolo dogs was trypsinized into peptides, washed, and sorted according to hydrophobicity with a C18 liquid chromatography column. The peptides were sorted according to mass/charge ratio in a mass spectrometer and the 10 most abundant precursor peaks of the MS1 spectrum selected for further analysis. The fragmentation spectra were filtered with an accepted false discovery rate of 5%. Peptides were identified based on canine proteins annotated in the Uniprot data base (https://www.uniprot.org/proteomes/UP000002254) using Proteome Discoverer software (ThermoFisher). Unreviewed canine proteins and the *ATG4D* variant c.1288G>A were included in the search. Proteins identified based on 2 or more specific peptides and with 2 or more peptide spectrum matches (PSM) were included in further analysis as outlined in Figure 1. Proteins present in 2 or 3 samples from affected dogs were grouped to represent the EV proteome of the affected group, and proteins present in 2 or 3 samples from controls were grouped to represent the EV proteome of the control group. The unique proteins of each group were determined as proteins present in 2 or all 3 samples of the group but absent in all 3 of the comparison group. FunRich software for functional enrichment and interaction network analysis of genes and proteins (http://www.funrich.org/) was used for qualitative analysis as well as comparison of the cellular compartment of origin and molecular function of the EV proteins. For protein-content-based EV characterization and quality control of the EV proteomes in general, proteins from categories 1, 2, and 3 (Table 3 in Théry et al^28^) were used as reference. The data analyzed in this study are available in Supplemental Materials (proteomic results) or upon request to the author (IEM quantification, NTA data).

## Results

### The Limiting Membranes of Accumulating Vesicles Contain Lysosomal, Autophagosomal, and Endosomal Proteins

**Figures 2–7.**
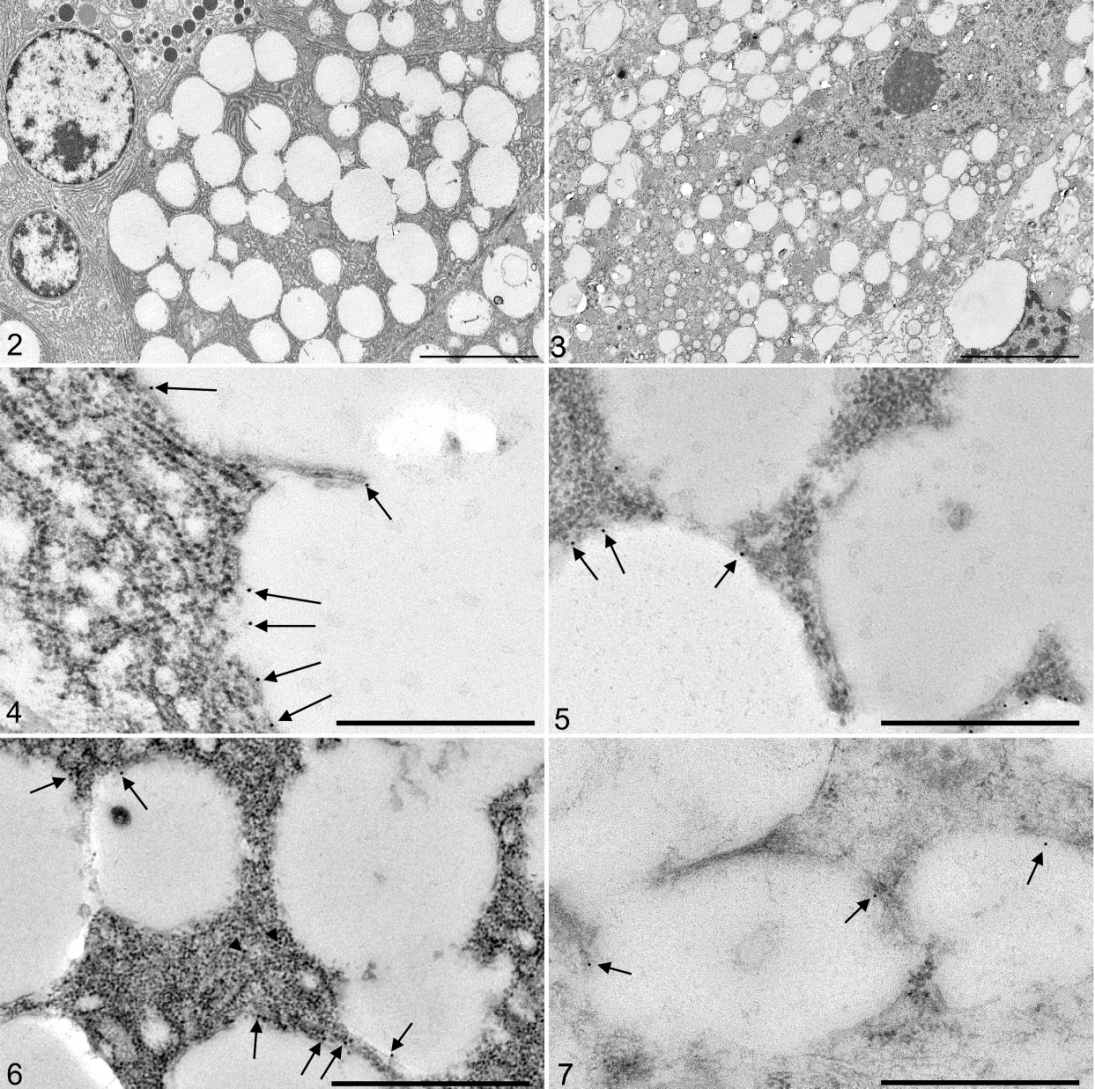
Lagotto Romagnolo dog with variant *ATG4D* gene. Case 1. Transmission electron microscopy (TEM) and immunoelectron microscopy (IEM). **Figure 2**. Pancreas. Acinar cells have numerous cytoplasmic membrane-bound clear vacuoles. TEM. Bar = 5 μm. **Figure 3**. Dorsal root ganglion (DRG). A neuron has extensive cytoplasmic vacuolization. IEM; isotype control antibody. Bar = 5 μm. **Figure 4**. Pancreas. Immunolabeling for LAMP2 protein by gold particles (arrows) on the limiting membranes of the cytoplasmic vacuoles. IEM for LAMP2. Bar = 500 nm. **Figure 5**. DRG. Gold particles (arrows) represent immunolabeling for ATG4D on the vacuolar limiting membrane. IEM for ATG4D. Bar = 500 nm. **Figure 6**. Pancreas. There is lower immuno-gold labeling density for LC3 on the vacuolar membrane (black arrows) and in the cytoplasm (arrowheads). IEM for LC3. Bar 500 nm. **Figure 7**. DRG. There is weak labeling for TFR2 on the vacuolar membrane. IEM for TFR2. Bar = 500 nm.

IEM was used to study the identity of the vacuoles accumulating in the affected tissues (Supplemental Figs. S1 and S2). The limiting membranes of the vacuoles that accumulated in the cells of one affected dog were characterized. The gold labeling density of lysosomal, autophagosomal, and endosomal proteins on the limiting membrane of cytoplasmic vacuoles in 2 affected organs (pancreas and DRG; Figs. 2, 3) was compared to the gold labeling density in sections of the same organs that were incubated with an isotype-control antibody and to those incubated without a primary antibody. The average membranous gold labeling density on the vacuolar membrane was 0.7 particles/μm^2^ in preparations without a primary antibody, and 3.2 particles/μm^2^ in preparations labeled with the isotype control antibody. The nonspecific membranous gold labeling density of 3.2 particles/μm^2^ was subtracted from the membranous gold labeling density in sections labelled with LAMP2, LC3, TFR2, and ATG4D. The remaining gold label on the vacuolar membranes was interpreted as specific. The quantitative assessment of the IEM gold label specificity, when corrected with the nonspecific labeling of isotype controls, is presented in Table 2.

**Table 2.** Quantitative Assessment of the Specificity of Immunoelectron Microscopic Gold Labelling on the Limiting Membrane of the Vesicles That Accumulate in the Pancreas and the Dorsal Root Ganglion of an Affected Lagotto Romagnolo Dog.

	Pancreas		Dorsal root ganglion	
Antigen	D(s)^a^, gold/μm^2^	F(s), %^b^	D(s), gold/µm^2^	F(s), %
LC3I/II	2.8	46*	3.1	46
TRF2	5.1	61	3.0	45
ATG4D	7.3	69*	3.2	47
LAMP2	21.9	95	14.3	93

^a^ D(s): specific membranous labeling density, defined as the total membranous density subtracted by the unspecific membranous density of isotype and negative controls.

^b^ F(s): specific labeling fraction, defined as % specific density of total density.

*P < .05 of χ^2^ when comparing membranous versus nonmembranous label distribution to the label distribution of the isotype control antibody.

IEM readily detected LAMP2 at the limiting membranes of the vacuoles that accumulate in both DRG neurons and pancreatic acinar cells (Fig. 4) with several-fold higher specific membranous labeling density than that of the nonspecific labeling (Table 2). The vacuolar limiting membrane was positively labeled for TFR2 and ATG4D in pancreas, with a weaker positive signal for these markers in the DRG (Figs. 5, 7). LC3 and ATG4D showed the weakest specific membranous labeling density (Table 2, Fig. 6). Both the LC3 and the ATG4D antibody bind to proteins that can locate on the autophagosomal membrane and be free in the cytoplasm. When comparing the labeling distribution (membranous vs cytoplasmic) of LC3 and of ATG4D with that of the isotype control antibody by the χ^2^ test, there was a statistically significant shift toward pancreatic membranous labeling in both markers, indicating that LC3 and ATG4D are part of the limiting membrane of the accumulating vacuoles. In conclusion, the limiting membrane of the vacuoles accumulating in the affected tissues contain lysosomal, endosomal, and autophagosomal proteins. Furthermore, ATG4D is localized to the limiting membrane of the accumulating vacuoles in Lagotto Romagnolo dogs with the *ATG4D* variant.

### Cells With the ATG4D Variant Release Increased Amounts of EVs Under Basal Conditions

**Figure 8.**
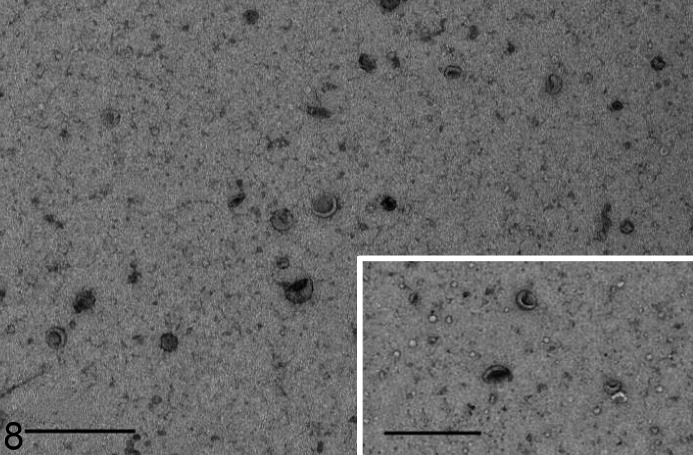
Extracellular vesicles in the culture medium of fibroblasts, Lagotto Romagnolo dog with variant *ATG4D* gene, case 3. The extracellular vesicles show the characteristic single membrane lining and cupped shape. Transmission electron microscopy, negative staining. Bar = 500 nm. Inset: Extracellular vesicles in the culture medium of fibroblasts from a control dog, case 4. Transmission electron microscopy. Negative staining. Bar 500 nm.

The concentration and size distribution of EVs released from fibroblasts of affected dogs and control dogs were compared. During basal conditions, the mean concentration of EVs in the medium of affected fibroblasts was significantly higher than that of control fibroblasts (Table 3). During starvation, the differences disappeared. The size distribution of EVs was not significantly different when comparing vesicles released from affected cells to those released by control cells, neither under basal conditions nor under starvation. Transmission electron microscopy confirmed the typical cup-shaped form of the EVs released from affected cells, as well as from control cells (Fig. 8).

**Table 3.** Concentration and Size Distribution of EVs Derived From Culture Medium of Fibroblasts From Affected and Control Lagotto Romagnolo Dogs.

	Exosome concentration (E10/ml)^a^; mean ± SEM		Particle size (nm); mean ± SEM	
Group	Full medium	Starvation	Full medium	Starvation
Affected *n* = 3	9.0 ± 0.76^*^	2.9 ± 0.48	203.4 ± 5.9	168.8 ± 7.9
Controls *n* = 3	6.7 ± 0.76	1.5 ± 0.48	195.5 ± 8.2	160.6 ± 6.5

*P < .05 when compared to control in linear mixed model.

^a^ E10/ml = ×10^10^ particles/ml.

### The EV Proteome of Fibroblasts With the ATG4D Variant Is Enriched With Chaperones During Basal Conditions

**Figures 9–12.**
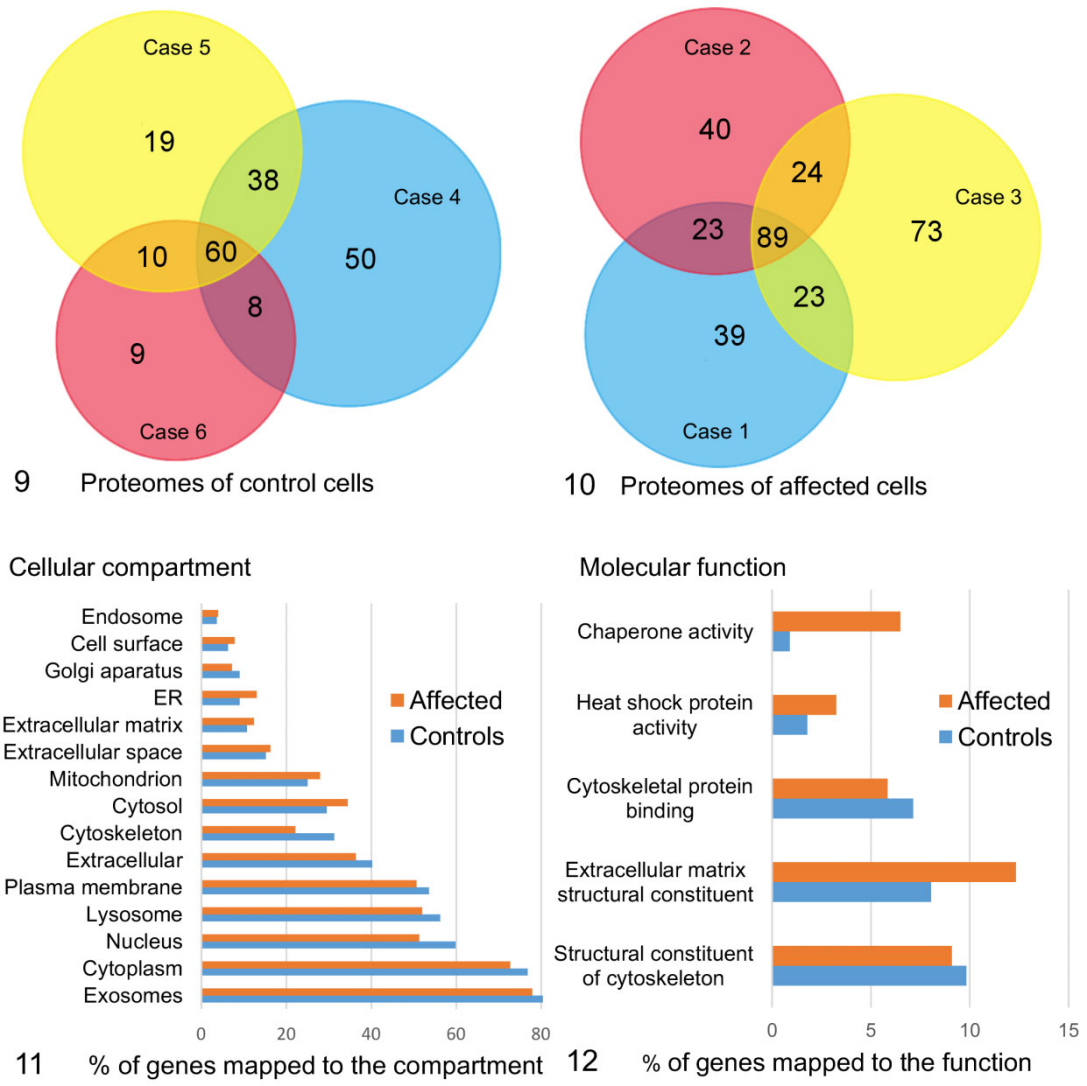
Quantitative and qualitative comparison of the extracellular vesicle (EV) proteomes of fibroblasts derived from 3 control and 3 affected Lagotto Romagnolo dogs. **Figure 9**. Proteomes of the EVs of control cells. **Figure 10**. Proteomes of the EVs of affected cells. **Figure 11**. Comparison of the predicted cellular origin of the proteins in EVs from control and affected cells. **Figure 12**. Comparison of predicted molecular functions of proteins in the EVs of control and affected cells.

Mass spectrometry was used to analyze the proteome of EVs released from control and affected fibroblasts. During basal conditions, the proteome of EVs from ATG4D mutant fibroblasts was more diverse than that of the control cells, with an average of 187 ± 20 different proteins, compared to an average of 124 ± 34 different proteins in the EV proteome of control cells (mean ± standard deviation). The EV proteomes of the control cells had 116 different proteins in common (Fig. 9), while the EV proteomes of affected cells had 159 different proteins in common (Fig. 10). Assessment of the cellular compartment of origin for these proteins revealed a shift toward those derived from the cytosol, mitochondria, and endoplasmic reticulum in the EV proteome of affected cells, with less proteins derived from the nucleus, lysosomes, and cytoskeleton than in the control cells (Fig. 11). When comparing the molecular function of the proteins, the EV proteome of mutant cells was enriched in proteins with chaperone activity and with extracellular matrix structural proteins (Fig. 12).

The proteins unique to control or mutant cells were identified. The unique proteins of the mutant cells included several chaperone proteins derived from endoplasmic reticulum or mitochondria (Table 4) as well as membranous and extracellular matrix proteins and proteins with enzymatic activity (Supplemental Table S1). Also among the unique proteins in the EVs of mutant cells was the major vault protein involved in transport, and clathrin heavy chain 1 that is the major protein of coated pits and vesicles. Among proteins unique to controls, 2 small GTPases active in vesicular transport were detected (Table 4). P62/sequestosome 1 was present in the EVs of all 3 mutant cases and none of the controls. However, p62 was detected based on one unique peptide in 2 cases (cases 1 and 2) and on 3 unique peptides only in case 3 and therefore was not included in the functional analysis. ATG4D was not detected in the EV proteomes of affected or control cells.

**Table 4.** Unique Proteins of the EV Proteome in Affected and Control Cells, Related to Protein Degradation, Vesicular Transport, and Release.

Molecular function	Mapped genes	Identified protein
Unique to affected		
Chaperone activity	CALR, CANX, HSPA5, TCP1, CCT3, CCT4, CCT5	Calreticulin, calnexin, heat-shock protein family A, T-complex protein 1 subunits, chaperonin containing TCP1
Nucleocytoplasmic transporter activity	MVP	Major vault protein
Heat shock protein activity	HSP90B1	Endoplasmin
GTPase activity	RHOA	Transforming protein RhoA
Calcium ion binding	ANXA4;	Annexin 4A
Structural molecule activity	CLTC;	Clathrin heavy chain
Cell adhesion molecule activity	FAT1;	FAT atypical cadherin1
Unique to controls		
GTPase activity	RAB1A, RAB5C	Ras-related proteins Rab1A and Rab5C
Protease inhibitor activity	SERPINC1	Antithrombin

The majority of proteins in the EV proteome of both ATG4D mutants (78%) and that of control cells (80%) mapped to proteins previously shown to locate to exosomes (Fig. 11). These proteins included transmembrane proteins or proteins anchored to the plasma membrane and/or endosomes (ie, Category 1 in Théry et al^28^) such as alpha and beta integrins (ITGA1, ITGA5, ITGB1), heparan sulfate proteoglycans, and CD44. In addition, a number of cytosolic proteins recovered in EVs (ie, Category 2 in Théry et al^28^) were present in the common proteome of both ATG4D mutant and control cells, including heat-shock protein family A member 8 (HSPA8), 90 alpha family class B member 1 (HSP90AB1), and annexins 1, 2, 5, and 6 (Supplemental Table S1). As an EV purity assessment, some proteins known to co-isolate with EVs despite deriving from non-EV structures (ie, Category 3 in Théry et al^28^) were used. APOA and APOB were detected in 5/6 samples, and albumin in 6/6 samples. The PSM values for APOA, APOB, and albumin were comparable between affected (median 6.2, range 3.6–11.6) and control samples (median 6.4, range 4.6–9.3). Furthermore, the PSM values were less abundant than those for annexins and integrins (affected median 41.1, range 33.2–49; control median 34.8, range 29.6–40). These results indicate that the EVs isolated from control and affected cells were equally pure.

## Discussion

Here we characterize the limiting membrane of the vacuoles accumulating in cells of Lagotto Romagnolo dogs with a mutant ATG4D gene and present the effect of altered basal autophagy on the amount and proteome of EVs released from affected cells. The vacuolar membrane contained the lysosomal marker LAMP2, expressed on late endosomes, conventional lysosomes, autolysosomes, and secretory lysosomes.^5^ LC3 was also detected on the limiting membrane, as was the endosomal marker TFR2. LC3 is present on autophagosomal membranes and phagocytic membranes during LC3-assisted phagocytosis.^18,22^ TFR2 is endocytosed during transferrin uptake and either recycled back to the plasma membrane through recycling endosomes or delivered via multivesicular bodies to the lysosome for degradation.^7^ Intracellular accumulation of vacuoles is a histopathological finding indicative of lysosomal storage diseases, where the vacuoles have been interpreted as secondary lysosomes containing partially or undegraded substrates. However, recent research has shown that the limiting membrane of the storage compartments can be more diverse, as is the case in mucopolysaccharidosis IIIB,^30^ where the membranes derive from the Golgi apparatus, and in multiple sulfatase deficiency and in mucolipidosis type II, where they represent compartments of the autophagy pathway, namely, autophagosomes and autolysosomes.^6,23^ The ATG4D-linked phenotype in Lagotto Romagnolo dogs adds to this group, as the main histopathological finding is accumulating intracellular vacuoles that are not solely of lysosomal origin. Despite a marker profile indicating that the vacuoles are hybrids between the endolysosomal and autophagosomal pathways, the morphology is inconsistent with that described for multivesicular bodies or amphisomes, as the vacuoles do not consistently contain intraluminal vesicles (ILVs) or sequestered degrading material.^9^

When assessed by IEM, ATG4D was detected on the membranes limiting the accumulating vacuoles with a labeling specificity and density comparable to the known membranous marker protein TFR2, and the autophagosome marker LC3. The subcellular location of ATG4D has not been reported, but functional studies on ATG4 in yeast^20^ and the human ATG4 paralogues A-D^1,11^ describe these cysteine proteases as transiently active on the autolysosomal membrane: first, revealing glycine on LC3 before lipidation, and second, during delipidation and release of LC3I from membrane-bound LC3II. The main role of ATG4D may not be in degradative, induced autophagy, as it is not required for this process.^11^ Instead, functional ATG4D has been linked to extracellular release of autophagic compartments^4^ and to caspase activation.^24^ Recently, it was shown in yeast that phospholipids on the autophagosomal membrane are not the sole target for ATG4-mediated LC3 conjugation, as ATG3 was described as an alternate target.^1^ Interestingly, ATG4 assists in conjugation of LC3 to ATG3 at the same site, which ATG12 uses in the ATG3-ATG12 complex. Notably, the ATG3-ATG12 complex is required for basal autophagy.^19^ This provides a potential link between ATG4 and functional basal autophagy at a molecular level, as altered ATG4D activity could influence ATG3-ATG12 formation and therefore affect basal autophagy.^1,19^

The quantitative and qualitative aspects of EVs and their content are increasingly being investigated also in veterinary medicine, especially within the field of companion animal medicine and veterinary oncology.^2,21^ Since EV isolation and characterization is very sensitive to the used methodology, constant efforts to standardize these aspects are made.^28^ Differential ultracentrifugation with wash is classified as a method of intermediate EV recovery and intermediate EV specificity^28^ and was applied in the current study as both quantitative and qualitative aspects of EVs release from LR fibroblasts were to be analyzed. It is possible that another EV isolation method could have revealed additional differences in the EVs released from fibroblasts of affected Lagotto Romagnolo dogs in comparison to those released from control cells. The EV concentration in media from LR control fibroblasts was, however, similar to the EV concentration previously reported in the culture medium of canine fibroblasts^2^ isolated by a different method, indicating that the chosen isolation method did not severely affect the quantitative EV aspects. The comparison of the EV proteome of fibroblasts from affected Lagotto Romagnolo dogs to breed-matched control cells, undergoing the same methodology as the affected cells, validate the qualitative differences. The EVs released by LR fibroblasts were overall larger than previously described canine fibroblast EVs; however, the results are based on only few dogs. In cells with mutant ATG4D, significantly more EVs were released during basal conditions in comparison to the controls. Increased release of EVs has been implicated as an alternate disposal mechanism and compensatory re-routing of undegraded material in cells with inhibited autophagosomal-lysosomal degradation,^8,31^ including rodent and human neuronal cells.^16^ It is possible that the accumulating vacuoles in affected cells are late autophagosomal or endosomal compartments, devoid of content and ILVs due to extracellular release of the same. Findings in fibroblasts cannot, however, directly be extrapolated to other cell types, and it remains to be investigated if the increased EV release and the enrichment of substrates of basal autophagy in EVs can be detected also in the serum or plasma of affected Lagotto Romagnolo dogs. The size spectrum of EVs in mutant cells did not differ significantly from that of control cells; hence, the ATG4D-variant does not affect the overall EV profile. Several chaperones, such as calreticulin, heat shock protein family A, and T-complex protein 1 subunits/CCTs, were exclusively present in the EV proteome of affected cells. Interestingly, Zhang and colleagues^32^ modelled dysfunctional basal autophagy in human fibroblasts and detected that the proteasome and T-complex protein 1 subunits/CCTs are substrates of basal autophagy. P62, which was present in the EV proteome of all affected cells and none of the controls, is a known substrate of autophagy.^32^ Our findings indicate that dysfunctional basal autophagy in cells with mutant ATG4D can redirect basal autophagy substrates into EVs. The small GTPases Ras-related proteins Rab1A and Rab5C were detected among the unique proteins of the control EV proteome, thus lacking in the EV proteome of affected cells. As the release of EVs is increased and the overall EV size profile not significantly different in affected cells, the lack of GTPases within the EVs may indicate that the cells with the mutant ATG4D retain the GTPases intracellularly. Rab GTPases are central regulators of membrane traffic within the autophagic, endosomal-lysosomal, and phagosomal pathways, with Rab1A and Rab5C mainly locating to the early endosomal compartment.^25^ Considering the cytoplasmic vacuolar tethering, fusion, engulfment, and inverted budding described in cells and tissues of Lagotto Romagnolo dogs affected by the vacuolar storage disease,^26^ the RAB proteins may be occupied in intracellular membrane traffic in cells with the ATG4D-mutation.

In conclusion, the limiting membrane of the vacuoles accumulating in the cells of diseased Lagotto Romagnolo dogs with mutant ATG4D contain proteins of the autophagosomal, endosomal, and lysosomal compartments, suggesting they may be hybrid organelles of these compartments. Fibroblasts from affected dogs release increased amounts of EVs during basal conditions and the accumulating vacuoles may contribute to this in a similar way as amphisomes or multivesicular bodies contribute to the EV release by fusing with the plasma membrane. During basal conditions, ATG4D mutant cells release EVs enriched with chaperones, some of which have been shown to be substrates of basal autophagy.

## Supplemental Material

Combined_supplemental_materials-Syrja_et_al - Altered Basal Autophagy Affects Extracellular Vesicle Release in Cells of Lagotto Romagnolo Dogs With a Variant *ATG4D* Click here for additional data file. Combined_supplemental_materials-Syrja_et_al for Altered Basal Autophagy Affects Extracellular Vesicle Release in Cells of Lagotto Romagnolo Dogs With a Variant *ATG4D* by Pernilla Syrjä, Mari Palviainen, Tarja Jokinen, Kaisa Kyöstilä, Hannes Lohi, Petra Roosje, Linda Anderegg, Tosso Leeb, Antti Sukura and Eeva-Liisa Eskelinen in Veterinary Pathology
