# Supplementary material for: Altered Basal Autophagy Affects Extracellular Vesicle Release in Cells of Lagotto Romagnolo Dogs With a Variant ATG4D
Source: Vet Pathol. 2020 Oct 5;57(6):926–35. doi: 10.1177/0300985820959243 (PMC7747043; doi:10.1177/0300985820959243)
Supplement: Combined_supplemental_materials-Syrja_et_al - Altered Basal Autophagy Affects Extracellular Vesicle Release in Cells of Lagotto Romagnolo Dogs With a Variant ATG4D [file Combined_supplemental_materials-Syrja_et_al.pdf]

Veterinary Pathology: Supplemental Materials.
Syrjä et al. Altered basal autophagy affects extracellular vesicle release in cells of Lagotto Romagnolo dogs with a variant *ATG4D*.

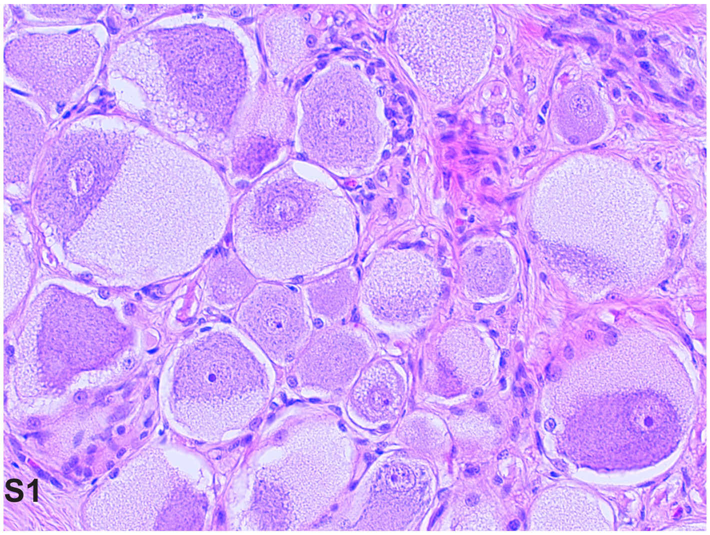


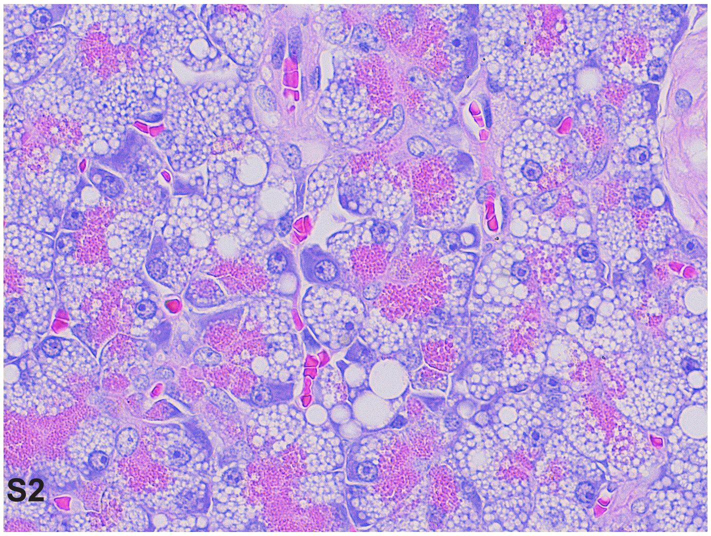


**Supplemental Figure S1**: Pancreas of an affected dog, showing diffuse, variably sized cytoplasmic vacuolization of the acinar cells of the exocrine pancreas. HE.

**Supplemental Figure S2**: Neurons in the dorsal root ganglium of an affected LR, with cytoplasm extended by fine vesicles and occasional larger vacuoles. HE.

Veterinary Pathology: Supplemental Materials.
Syrjä et al. Altered basal autophagy affects extracellular vesicle release in cells of Lagotto Romagnolo dogs with a variant *ATG4D*.

**Supplemental Table S1: MOLECULAR FUNCTION OF PROTEINS IDENTIFIED IN THE EV PROTEOME**

| A. PROTEINS COMMON TO AFFECTED AND CONTROLCELLS | | B. PROTEINS UNIQUE TO CONTROL CELLS | | C. PROTEINS UNIQUE TO AFFECTED CELLS | |
|---|---|---|---|---|---|
| Dataset: control and affects | | Dataset: UniqueControls | | Dataset: Unique Affects | |
| Enrichment analysis: Molecular function | | Enrichment analysis: Molecular function | | Enrichment analysis: Molecular function | |
| Number of genes in the dataset: 85 | | Number of genes in the dataset: 14 | | Number of genes in the dataset: 41 | |
| Number of genes in the dataset (which are available in Molecular function database) :85 | | Number of genes in the dataset (which are available in Molecular function database) :14 | | Number of genes in the dataset (which are available in Molecular function database) :41 | |
| | | | | | |
| | | | | | |
| **Molecular function** | **Mapped gene names** | **Molecular function** | **Mapped gene names** | **Molecular function** | **Mapped gene names** |
| Extracellular matrix structural constituent | COL12A1; COL1A1; COL1A2; EDIL3; FBLN1; FN1; THBS1; THBS2; VCAN; | Structural constituent of cytoskeleton | ACTB; ACTC1; KRT14; | Chaperone activity | CALR; CANX; CCT3; CCT4; CCT5; HSPA5; PPIB; TCP1; |
| Structural constituent of cytoskeleton | ACTN4; CTNNA1; EPB41L2; GSN; MSN; TUBA1A; TUBB; VIM; | Channel regulator activity | STOM; | Extracellular matrix structural constituent | COL3A1; COL5A1; COL5A2; LAMA2; LAMB1; LAMB2; LAMC1; NID1; |
| Cytoskeletal protein binding | ACTN1; CALD1; CFL1; FLNC; TLN1; VASP; VCL; | GTPase activity | RAB1A; RAB5C; | Structural constituent of cytoskeleton | ACTR1A; ACTR3; TUBB6; |
| Structural molecule activity | AFM; KRT1; KRT10; KRT5; MYH9; MYO1D; PLS3; | Complement activity | CFB; | Nucleocytoplasmic transporter activity | MVP; |
| Calcium ion binding | ANXA1; ANXA2; ANXA5; ANXA6; CALM1; | Protease inhibitor activity | SERPINC1; | Isomerase activity | P4HB; PDIA3; |
| Catalytic activity | CKB; GAPDH; LDHA; LOXL2; PGK1; PGM1; PHGDH; PKM; | Oxidoreductase activity | CYBRD1; | Ligase activity | AARS; ASNS; |
| Cell adhesion molecule activity | CDH13; ICAM1; MFGE8; TNC; TNS1; ZYX; | Structural molecule activity | KRT75; | Structural constituent of ribosome | RPLP2; RPS26; |
| Heat shock protein activity | CRYAB; HSPA8; | Cell adhesion molecule activity | ITGB5; | Heat shock protein activity | HSP90B1; |
| Intracellular ligand-gated ion channel activity | CLIC1; CLIC4; | Catalytic activity | LOXL4; | Protein binding | CSPG4; PTGFRN; |
| ATPase activity | ATP1A1; ATP2B4; DYNC1H1; | Transporter activity | FETUB; | Protein serine/threonine phosphatase activity | PPP2R1A; |
| Receptor activity | CD44; ITGA1; ITGA5; ITGAV; ITGB1; | DNA binding | HMGB1; | Peptidase activity | PLG; |
| Kinase activity | PKM; | | | Serine-type peptidase activity | FAP; |
| Peroxidase activity | PRDX1; | | | Calcium ion binding | ANXA4; |
| Lyase activity | ALDOA; | | | GTPase activity | RHOA; |
| Cytoskeletal anchoring activity | FLNA; | | | Structural molecule activity | CLTC; |
| Defense/immunity protein activity | AHSG; | | | Cell adhesion molecule activity | FAT1; |
| Transporter activity | ALB; APOA1; APOB; VAT1; | | | Receptor activity | PVR; |
| Motor activity | MYO1C; | | | Catalytic activity | MTHFD1; |
| Protease inhibitor activity | A2M; | | | DNA binding | HIST2H2AC; |
| Translation regulator activity | EEF2; | | | Molecular function unknown | ARHGDIA; CYFIP1; |
| Ligase activity | GARS; | | | | |
| Receptor signaling complex scaffold activity | YWHAH; YWHAZ; | | | | |
| Chaperone activity | HSP90AB1; | | | | |
| Receptor binding | LGALS1; | | | | |
| Protein binding | AHNAK; | | | | |
| Hydrolase activity | DPYSL3; | | | | |
| Auxiliary transport protein activity | SLC1A5; | | | | |
| Transcription regulator activity | TGFB1I1; | | | | |
| Molecular function unknown | CAP1; LPP; MYOF; | | | | |
